# Correction: Domrazek, K.; Jurka, P. Application of Next-Generation Sequencing (NGS) Techniques for Selected Companion Animals. *Animals* 2024, *14*, 1578

**DOI:** 10.3390/ani15040606

**Published:** 2025-02-19

**Authors:** Kinga Domrazek, Piotr Jurka

**Affiliations:** Institute of Veterinary Medicine, Warsaw University of Life Sciences—SGGW, Nowoursynowska 159c, 02-776 Warsaw, Poland; piotr_jurka@sggw.edu.pl

The authors would like to make the following corrections in the original publication [1]. The changes are as follows:

(1) Replacing the following sentence in “Section 5. Veterinary Parasitology”.

“Huggings et al. developed and validated a long-read metabarcoding platform for the detection of filarial worm pathogens of animals and humans [68]. They demonstrated that NGS could be a specific and sensitive method for diagnosing filarial worms, but on the other hand, they also showed that this method is not ideal. In one case, genomic tools showed false-negative results [68]. This method might be refined through further research”.

with

“Kattor et al. used Targeted Next-Generation Sequencing for comprehensive testing for selected vector-borne pathogens in canines [68]. Their results showed false-negative results in two samples [68]. This method might be refined through further research”.

(2) Replacing the reference [68].

68. Huggins, L.G.; Atapattu, U.; Young, N.D.; Traub, R.J.; Colella, V. Development and Validation of a Long-Read Metabarcoding Platform for the Detection of Filarial Worm Pathogens of Animals and Humans. *BMC Microbiol.* **2024**, *24*, 28.

with

68. Kattoor, J.J.; Nikolai, E.; Qurollo, B.; Wilkes, R.P. Targeted Next-Generation Sequencing for Comprehensive Testing for Selected Vector-Borne Pathogens in Canines. *Pathogens* **2022**, *11*, 964. https://doi.org/10.3390/pathogens11090964.

The authors state that the scientific conclusions are unaffected. This correction was approved by the Academic Editor. The original publication has also been updated.
